# Sexually Dimorphic Genome-Wide Binding of Retinoid X Receptor alpha (RXRα) Determines Male-Female Differences in the Expression of Hepatic Lipid Processing Genes in Mice

**DOI:** 10.1371/journal.pone.0071538

**Published:** 2013-08-19

**Authors:** Astrid Kosters, Deqiang Sun, Hao Wu, Feng Tian, Julio C. Felix, Wei Li, Saul J. Karpen

**Affiliations:** 1 Division of Gastroenterology, Hepatology, and Nutrition, Department of Pediatrics, Emory University School of Medicine, Atlanta, Georgia, United States of America; 2 Division of Biostatistics, Dan L. Duncan Cancer Center, Department of Molecular and Cellular Biology, Baylor College of Medicine, Houston, Texas, United States of America; 3 Department of Biostatistics, School of Public Health, Emory University, Atlanta, Georgia, United States of America; 4 Division of Gastroenterology, Hepatology, and Nutrition, Department of Pediatrics, Baylor College of Medicine, Houston, Texas, United States of America; Beckman Research Institute of City of Hope, United States of America

## Abstract

Many hepatic functions including lipid metabolism, drug metabolism, and inflammatory responses are regulated in a sex-specific manner due to distinct patterns of hepatic gene expression between males and females. Regulation for the majority of these genes is under control of Nuclear Receptors (NRs). Retinoid X Receptor alpha (RXRα) is an obligate partner for multiple NRs and considered a master regulator of hepatic gene expression, yet the full extent of RXRα chromatin binding in male and female livers is unclear. ChIP-Seq analysis of RXRα and RNA Polymerase2 (Pol2) binding was performed livers of both genders and combined with microarray analysis. Mice were gavage-fed with the RXR ligand LG268 for 5 days (30 mg/kg/day) and RXRα-binding and RNA levels were determined by ChIP-qPCR and qPCR, respectively. ChIP-Seq revealed 47,845 (male) and 46,877 (female) RXRα binding sites (BS), associated with ∼12,700 unique genes in livers of both genders, with 91% shared between sexes. RXRα-binding showed significant enrichment for 2227 and 1498 unique genes in male and female livers, respectively. Correlating RXRα binding strength with Pol2-binding revealed 44 genes being male-dominant and 43 female-dominant, many previously unknown to be sexually-dimorphic. Surprisingly, genes fundamental to lipid metabolism, including Scd1, Fasn, Elovl6, and Pnpla3-implicated in Fatty Liver Disease pathogenesis, were predominant in females. RXRα activation using LG268 confirmed RXRα-binding was 2–3 fold increased in female livers at multiple newly identified RXRα BS including for Pnpla3 and Elovl6, with corresponding ∼10-fold and ∼2-fold increases in Pnpla3 and Elovl6 RNA respectively in LG268-treated female livers, supporting a role for RXRα regulation of sexually-dimorphic responses for these genes. RXRα appears to be one of the most widely distributed transcriptional regulators in mouse liver and is engaged in determining sexually-dimorphic expression of key lipid-processing genes, suggesting novel gender- and gene-specific responses to NR-based treatments for lipid-related liver diseases.

## Introduction

The nuclear receptor (NR) Retinoic X Receptor (RXRα; NR2B1) is the obligate heterodimerization partner for >14 Class II NRs including: LXRα,β (NR1H2,3), PPARα β,γ (NR1C1–3), RARα β,γ (NR1B1–3), FXR (NR1H4), PXR (NR1I2), TR (NR1A1) and VDR (NR1I1) [1]. NRs are ligand activated transcription factors and RXRαis activated by 9-cis retinoic acid and other lipids, as well as by several synthetic ligands (e.g. LG268) [2]. DNA binding sites of Class II NRs consist of 2 half-site motifs based upon the consensus sequence AGGTCA, with variation in the number of nucleotides spaced between the 2 half-sites (from 0–8) that helps direct heterodimer binding affinities. Furthermore, the functionality of RXRα:partner heterodimerization complexes can be categorized as *permissive* (such as FXR, LXRα and PPARα β,γ) or *non-permissive* (such as RARα and VDR), depending upon their responses to ligands for each partner [1]. Activation of *permissive* heterodimers occurs by ligands for both RXRα and its partner, either independently or together, the latter results in synergistic activation of gene transcription, whereas activation of *non-permissive* RXRα heterodimers occurs through ligand binding of the NR-partner and is unaffected by the presence of an RXRα agonist.

RXRα is primarily expressed in the liver, as well as in many other tissues at lower level, including kidney and gut [3]. The liver plays an essential and often biologically exclusive role in multiple physiological processes including lipid and xenobiotic metabolism, bile formation, nutrient handling, many of whose core effector genes are regulated by RXRα-containing heterodimers. As the obligate partner, RXRα is central to the regulation of liver gene expression, and its actions are often considered as driven through activation of its multiple permissive partners. The observation that a hepatocyte-specific deletion of the DNA-Binding Domain (DBD) of RXRα in mice resulted in changes in the expression of genes from multiple pathways in the liver, emphasizes the central and pleiotropic role of RXRα and confirms RXRα integrates multiple physiological processes in the liver [4]–[6].

The existence of gender differences in liver biology has been reported in multiple studies and involves a wide range of hepatic functions [7]–[10] and sexual dimorphism in hepatic gene expression has been shown for more than 1000 genes in both rats and mice [11]–[13]. Differential patterns of Growth Hormone (GH) release from the pituitary between males and females is one of the principal determinants of sex differences in hepatic gene expression [14], [15]. GH downstream signaling in hepatocytes is mediated by the transcription factor Stat5b and deletion of Stat5b led to a loss of liver sexual dimorphism with down-regulation of approximately 90% of male-specific genes in male mice [11], [16], making Stat5b a primary regulator of hepatic sex-specific gene expression [17]. Besides Stat5b, other transcription factors have been identified in regulating hepatic sexually dimorphic gene expression, including Blc6 [18], HNF6 (Onecut1) and Cux2 (Cutl2) [18]. In addition, roles for sex hormones as well as NRs e.g. HNF4α;[19], PPARα [9], [20] and GR [21] have been reported. However, a full regulatory assessment of hepatic sexually-dimorphic gene expression is not entirely explained by differences in GH, sex hormones and the above mentioned transcription factors. Moreover, many hepatic Phase I and Phase II drug-metabolizing and lipid-processing genes are differentially-expressed in males and females, some of these were identified as Stat5b-independent [11], [16], [22], and this may underlie gender-specific therapeutic drug responses, consequences of hepatic steatosis, and some adverse drug events in humans [23]–[26]. The majority of these genes are regulated by RXRα heterodimers, yet without a complete delineation of RXRα binding in male and female liver chromatin, knowledge of the underlying contributions of RXRα heterodimers to these pathways remains incomplete and impairs our ability to counter some of these clinically-relevant issues. To begin to close this gap, we sought to determine if sexually-distinct patterns of RXRα chromatin binding in liver would support its role as a relevant regulator of hepatic sexually-dimorphic gene expression of drug metabolizing and lipid processing genes.

## Materials and Methods

### Animals and Treatment

For ChIP-Seq analyses, livers were collected from eight-week-old male and female mice (n = 2/gender). Gavage feeding of LG268 (30/mg/kg/day) for 5 days was performed as described before [6]. Animal protocols and procedures were approved by the Institutional Animal Care and Use Committee Center (IACUC) at Baylor College of Medicine under protocol number AN-2024. During all procedures all efforts were made to minimize suffering of the mice.

### Nuclear extraction and Western blot analysis

Procedures were performed as described before [6], [27] and involved n = 4–6 per group.

### Chromatin Immunoprecipitation-sequencing (ChIP-Seq)

For Chromatin Immunoprecipitation for Sequencing, flash-frozen tissues were submitted to Genpathway (San Diego,CA) and was performed according to their FactorPath protocol [28]. ChIP-quality anti-RXRα antibody (D-20,sc-774X, Santa Cruz Biotechnology, Santa Cruz, CA) [29] and RNA Pol2 (ab24758, Abcam) were used. DNA libraries were sequenced on a Genome Analyzer II by Illumina Sequencing Services.

### Sequence data and ChIP-seq peak calling

RXRα and Pol2 ChIP-Seq reads were processed through Genome Analyzer Pipeline Software (Illumina) and aligned to mouse reference genome mm9 by ELAND with at most 2 mismatches. Reads that appeared more than twice at the same position on the same strand were discarded to remove PCR duplication. MACS [30] was used to identify RXRαpeaks at p-value cutoff E-10.

In addition, male peaks and female peaks overlapping by at least 1bp were considered “common” to both genders, otherwise both of these two peaks would be considered “unique”.

For each RXRα peak summit, the number of reads in both male and female samples was counted in a 300bp region centered on the peak summit, and a p-value evaluating gender difference was calculated assuming a Poisson distribution, which was then transformed to a score, which is in fact a signed and scaled p-value, defined as log2(−10*log10(p)) for stronger male peaks or as −log2(−10*log10(p)) for stronger female peaks. A score >6 was considered significantly different for male specific peaks, whereas a score <−6 was significantly different for female specific peaks.

For Pol2, the number of reads in the region from transcription starting site to transcription terminating site for each gene was counted in male and female samples, and calculated the Pol2 score for each gene in a similar way as for the RXRα peak score. BED and Wiggle files are stored in the UCSC database (http:// genome.ucsc.edu/) and can be downloaded using UCSC genome browser. In addition data are submitted to the NCBI Gene Expression Omnibus (GEO) database under GSE21696.

### Validation of ChIP-seq by ChIP-qPCR

Our ChIP-qPCR protocol was modified based on the EZ-ChIP kit protocol from Upstate Biotechnology (Upstate Biotechnology, Lake Placid, NY) and based on the UC-Davis lab protocol [31]. Briefly 300 mg finely crushed mouse liver tissue powder was crosslinked with formaldehyde at 1% final concentration. The reaction was stopped by addition of glycine (125 mM final concentration). 30 µg of sonicated chromatin (Bioruptor 300;Diagenode) was pre-cleared with protein-A agarose 50% slurry (Millipore). RXRα-bound genomic DNA regions were isolated using an RXRα antibody (see above) and incubated overnight at 4°C. Nonimmune rabbit IgG was used as negative control. Immune complexes were precipitated with protein-A agarose beads. Protein–DNA cross-links were reversed overnight at 65°C. DNA was purified using PCR purification columns (Qiagen,Valencia, CA). Levels of transcription factor occupancies at chromatin were determined by Realtime quantitative PCR (SYBrGreen, Applied Biosystems). Input and immunoprecipitated DNA reactions were performed in duplicate. Fold differences relative to control were expressed as mean±SEM. Primer sequences are available upon request.

### Microarray data analysis and validation by qRT-PCR

Microarray hybridization, analysis and normalization on mouse male and female liver using Ilumina Ref6v2 was performed by the core facility of Children's' Nutrition Research Center at Baylor College of Medicine, and data was submitted to the GEO database under number GSE21696. RNA isolation, cDNA and quantitative realtime PCR was performed as described before [6], [27]. Primer sequences are available upon request.

Comparison of ChIP-seq data with Microarray Gene Expression was performed using Microsoft Access database and Venn diagram generator [32]. Functional Annotation by Ontology and pathway analysis was performed using DAVID [33].

## Results

### Whole genome and sexually dimorphic RXRα binding in mouse liver chromatin by ChIP-Seq

**Figure 1:**
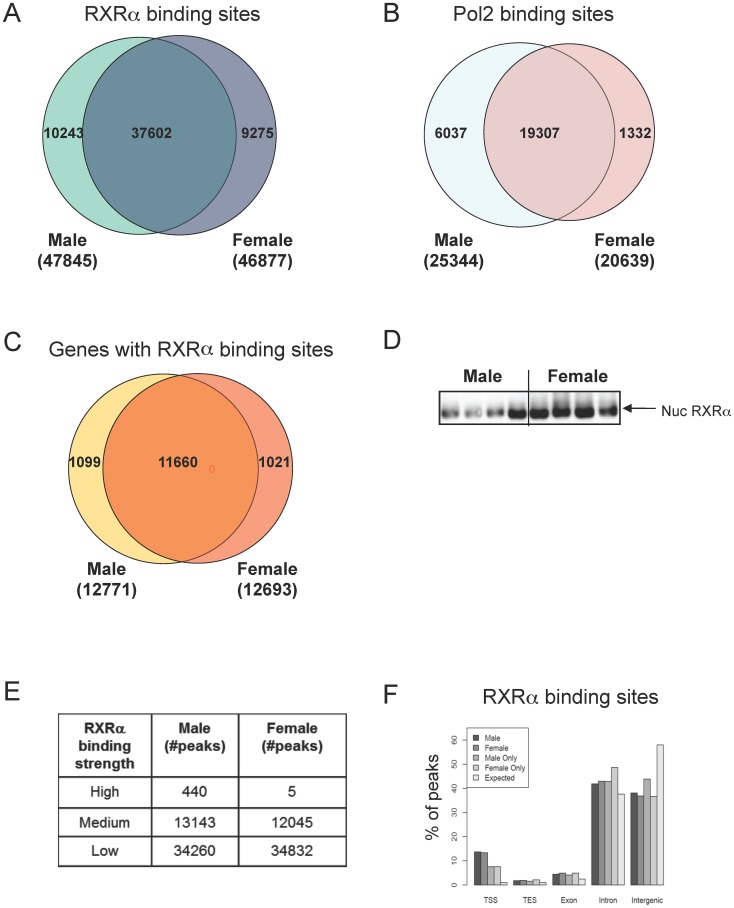
Genome wide mapping of RXRα and Pol2 binding sites in male and female mouse liver. A) Venn diagrams show number of RXRα binding sites in male and female livers with 37602 binding sites shared between genders, and 10243 unique RXRα male and 9275 unique RXRα female binding sites (see also Table S1A and S1B in File S1). B) Venn diagrams show number of Pol2 binding sites in male and female livers, with 19307 binding sites shared between genders, and 6037 unique male and 1332 unique female Pol2 binding sites. C) Venn diagrams representing number of genes associated with RXRα peaks shown in A), with 11660 genes shared between genders, and 1099 unique genes for male and 1021 genes for female. D) Western blot analysis shows increased nuclear RXRα protein levels in female mouse liver compared to male mouse liver. E) Number of RXRα peaks as stratified by peak height (see also Table S2A and S2B in File S1). F) Genomic positions of RXRα binding sites in male and female mouse liver. Data expressed as percentage of total peaks per gender. RXRα binding was significantly increased at TSS (+/−500 bp), TES, within exons and introns with a depletion in binding compared to the expected number of sites based on random distribution (see also Fig. S1A and S1B).

Both male and female mice were used to determine genome-wide RXRα binding with ChIP-Seq and thus explore potential roles for RXRα in gender-specific liver biology. ChIP-Seq analysis revealed 47,845 RXR α α binding sites in male mouse liver and 46,877 in females, of which 37,602 (∼80%) were common, leaving 10,243 and 9,275 sites unique in male and female livers respectively (Fig. 1A; Table S1A in File S1). To determine transcriptional activity correlating with RXRα binding, ChIP-Seq analysis for Pol2 was performed, which revealed 25,344 active sites in males and 20,639 sites in females, with an overlap of 19,307 sites (Fig. 1B, Table S1A in File S1). The overall greater male Pol2 binding was reflected by an asymmetric and substantial male-specific representation (6,037 male-specific vs. 1,332 female-specific).

There were nearly an equal number of genes in both genders (12,771 male and 12,693 female) associated with RXRα binding sites, with the vast majority (11,660/∼92%) in common (Fig. 1C). Overall, this indicates an average of ∼3–4 RXRα binding sites in each of the >12,500 expressed and potentially RXRα-regulated genes in mouse liver. Taken together, this large number of common and gender-specific RXRα binding sites in male and female livers (>45,0000) spread across ∼12,000 genes makes RXRα, to our knowledge, one of the most broadly distributed transcription factor in mouse liver chromatin found to date. No significant differences in RXRα binding site geographic distribution profiles or in chromosomal distribution for RXRα and Pol2 binding sites were noted between genders (Fig. S1A and S1B).

RXRα RNA levels are highest in liver compared to other mouse tissues [3], yet it was unknown if RXRα nuclear protein levels were differentially represented in male and female mouse livers, which could impact upon the differential binding. Surprisingly, even though there are slightly more RXRα binding sites in male compared to female liver chromatin, soluble nuclear quantities of RXRα were substantially greater in female than in male liver nuclei (Fig. 1D). These studies indicate that the genome-wide ChIP-Seq dataset, rather than the absolute levels detected by immunoblot, more accurately inform the potential regulatory roles played by RXRα in mouse liver.

With the large number of binding sites found, it was imperative to develop workable definitions to focus on those RXRα sites most likely involved in regulation of hepatic gene expression. First we looked at peak height as a measure of RXRα binding strength, and peak height distribution was arbitrarily divided as described in methods. Fig. 1E shows that for 440 male peaks (401 genes) the peak count was higher than 300 and this was the case for only 5 peaks in female mice (5 genes) (Tables S1B and S1C in File S1), suggesting a group of sites with overall stronger binding of RXRα in male liver compared to female liver. This is in contrast to expectations based on the higher nuclear protein levels of RXRα in female mice as shown on western blot in Fig. 1D.

As has been noted in other transcription factor ChIP-Seq analyses, a minor fraction (13%) of RXRα binding sites was located within 500 bp of the Transcription Start Site (TSS), while a substantial proportion (42%) bound in intronic regions and intergenic (38%) regions (Fig. 1F, Table S1D in File S1).

**Figure 2:**
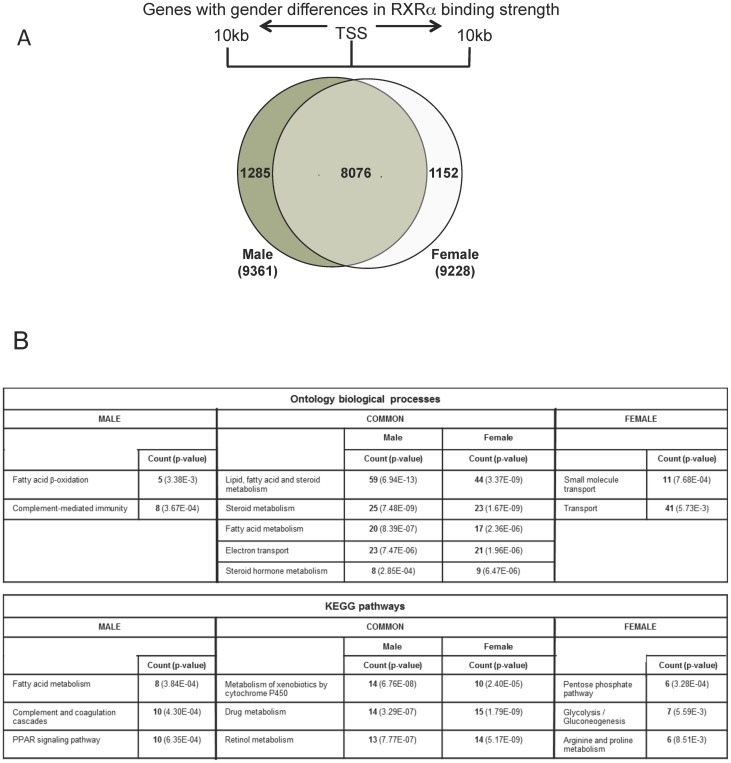
RXRα and Pol2 binding sites in mouse liver 10 kB surrounding the Transcriptional Start Sites. A) Venn diagram showing number of genes with shared RXRα regulation between male and female within 10 kB up and downstream of TSS, with 1285 genes found in male only and 1152 regulated by RXRα in females (see also Table S3A–D in File S1). B) Ontology analysis and pathway analysis of RXRα peaks with score >6 or <−6 located within +/−10 kB of TSS (see also Table S3E–F in File S1).

RXRα binding sites present within the span from 10 kb upstream to 10 kb downstream of the TSS were considered more likely to be “promoter and enhancer regions” and contain the most common gene regulatory regions involved in direct regulation of gene transcription. With this delineation, 16,898 RXRα binding sites in male liver (associated with 9,361 genes), and 15,870 RXRα binding sites (associated with 9,228 genes) in female liver were identified. The majority (8076) of these genes were represented in both genders, with 1285 specific for male liver and 1152 for female (Fig. 2A). Further exploration of these highly sexually-differential RXRα binding occupancies ensued to see if they uncovered new sexually-regulated genes.

### Ontology and pathway analyses

Ontology and pathway analysis of these RXRα-bound genes showed that a number of biological pathways were commonly represented in both genders, including lipid and steroid metabolism; however the genes within the pathways were not identical between genders (Fig. 2B). Gender-specific ontological functions and pathways included fatty acid metabolism and complement-mediated immunity and coagulation for male livers and transport function and glucose metabolic pathways in female mice (Fig. 2B; Table S2A–B in File S1).

### Graphic representation of RXRα-binding linked with Pol2 gene occupancy – the “RXRα+Pol2 Score”

Functional assignment of the potential relevance of RXRα binding is predicated upon whether or not the associated gene is transcribed by Pol2. To initially explore such assignments, prioritization was determined for each gene by incorporating a score for both RXRα-binding and Pol2-binding strengths, based upon RXRα peak height and the integration of Pol2 binding peak heights over the length of the gene (see Methods). By comparing RXRα peak height at each binding site between genders, and linking these gender-ranked RXRα binding strength scores to Pol2 occupancy of the gene in question, an RXRα+Pol2 scoring classification was established. This score was used to identify binding sites that differed, or were common, in male and female livers with strengths stratified as High (>6), Medium (3–6) or Low (<3) (Table 1). Of the genes with the strongest significant differences in RXRα peak height between genders (those with a score >6), 1945 genes exhibited male-specific RXRα binding (associated with 2996 RXRα binding sites), and 1214 genes were strongly associated with 1689 female-specific RXRα peaks. There were 283 genes (with 718 gender-dispersed strong RXRα peaks) common to both genders (Table 2, Table S3A–E in File S1). Of the strongest differences in Pol2 binding sites, 528 RXRα peaks were associated with 72 genes with a Pol2 score>6 in male livers, while for females 82 genes had a Pol2 score >6, with an associated 602 RXRα peaks (Table 2, Table S3F–G in File S1).

**Table 1:** Stratification of RXRα binding sites in male and female mouse liver according to binding.

RXRα -peak score	Male#peaks	Female#peaks
High >6	3428	2140
Medium 3–6	17842	16793
Low 0–3	26586	27932

Pol2 score	Male # RXRα peaks	Female#RXRα peaks
High >6	528	602
Medium 3–6	3987	2949
Low 0–3	43327	43315

**Table 2:** Stratification of RXRα binding sites in male and female mouse liver according to peak score.

	Mal#peaks	Female#peaks
**All RXRα peaks >6**	3428	2140
**Unique genes**	2227	1498
**Gender specific RXR**α **peaks >6**	2996	1689
**Gender specific unique genes**	1945	1214
**Overlap RXR**α **peaks >6**	718	718
**Unique genes**	283	283
**Gender specific Pol2 peaks >6**	528	602
**Gender specific unique genes**	72	82

**Figure 3:**
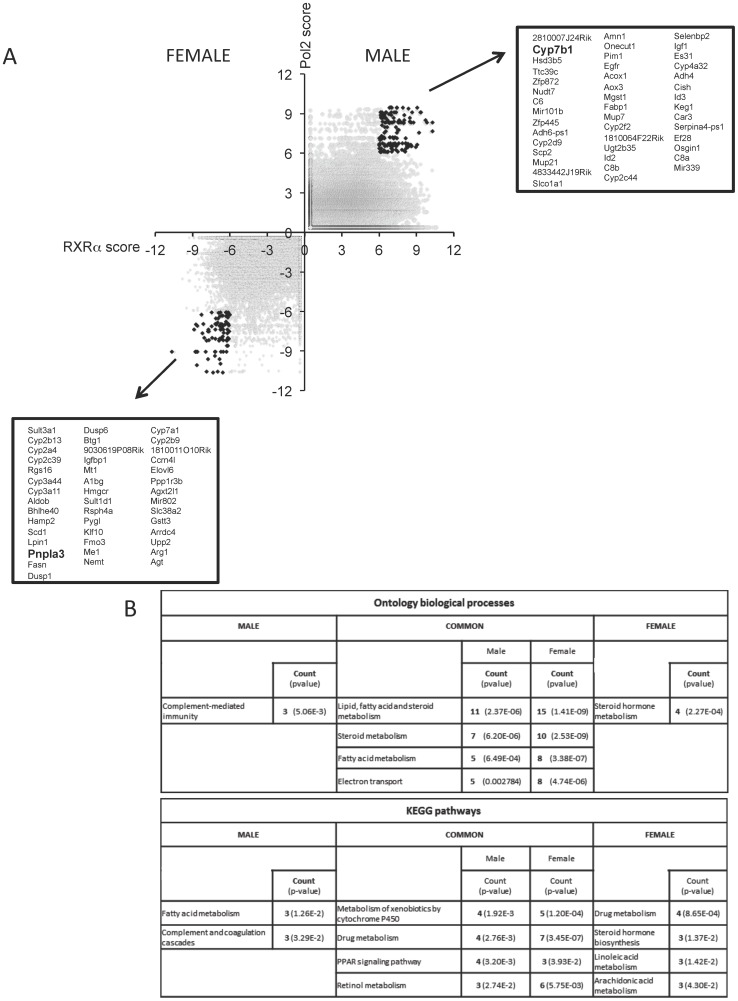
Sexual dimorphic RXRα-dependent gene regulation in mouse liver. A) Scatter plot of RXRα scores correlating with Pol2 scores. Genes identified having a RXRα and Pol2 score >6 for male and -<6 for females are represented by the black dots, with the grey dots representing correlation of all genes. Genes listed in order of RXRα score high to low (see also Table S5A and S5B in File S1). B) Ontology analysis and pathway analysis of genes with an RXRα and Pol2 score >6 for male and -<6 for females (see also Table S5C and S5D in File S1)

Combining all significant gender-specific RXRα-binding and Pol2-binding scores identified those sexually-dimorphic transcriptionally active genes with sexually-dimorphic RXRα binding. After applying these criteria for strong RXRα and Pol2 binding (RXRα>6 & Pol2>6), we found that male mouse liver chromatin contained 146 RXRα peaks associated with 44 male-specific genes while in female livers, 86 RXRα peaks correlated with 43 female genes (0.001% of total genes). This is represented in graphic form (Fig. 3A) with the gender-enriched gene names listed alongside the graph (see Table S4A–B in File S1for scores). Altogether, this new analysis provides a window into RXRα-dependent transcriptional competencies unique to male and female livers. Both individual gene evaluation and ontological pathway [34] analyses revealed an enrichment of genes in sterol, metabolic (lipid & glucose) and phase I & II detoxification pathways (Fig. 3B; Table S4C–D in File S1). Among the male-specific genes identified with this analysis were some that were previously determined to be male-enriched (e.g. Cyp7b1, Hsd3b5) but many that were not previously recognized (e.g. Ttc39c, Egfr, 2810007J24Rik). Similarly, in female livers, Sult3a1 and members of the Cyp2 and Cyp3 families were known to be female-dominant, but several others, especially those engaged in core sterol and lipid processing pathways were unexpectedly enriched. These include Scd1, Fasn, Pnpla3, Elovl6, Rgs16 and Lpin1. Of note, several of these genes have polymorphisms strongly linked to altered metabolic physiological outcomes, specifically related to obesity and lipid handling [35]–[43]. It was this unexpected limited roster of genes enriched in sterol and lipid processing pathways, identified by employing the combined RXRα+Pol2 scoring that led to further explorations of the role of RXRα in the expression of these key metabolic genes.

### Representative ChIP-Seq maps of sentinel sexually-dimorphic genes

**Figure 4:**
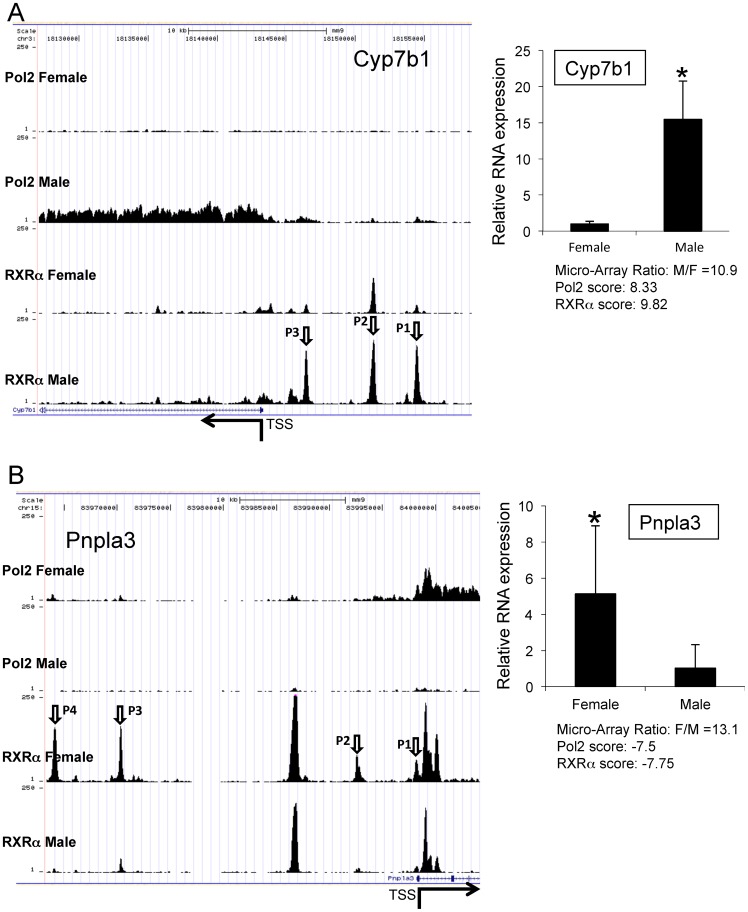
Representatives of gender differential RXRαregulated genes. A) Predominant RXRα and Pol2 binding for Cyp7b1 in male mouse liver. Left-hand panel: Top 2 tracks show Pol2 binding along the Cyp7b1 gene-span in male and female mouse liver. Bottom 2 tracks show RXRα peaks binding upstream of the TSS of Cyp7b1 TSS. Male enriched RXRα peaks are indicated by arrows P1, P2 and P3. Right-hand panel: Sexual dimorphic RNA levels of Cyp7b1 were confirmed by qPCR (n = 5–6) (see also Fig. S2 and S3A–G). B) Predominant RXRα and Pol2 binding for Pnpla3 in female mouse liver. Left-hand panel: Top 2 tracks show Pol2 binding along the Pnpla3 gene-span in male and female mouse liver. Bottom 2 tracks show RXRα peaks binding upstream of the TSS of Pnpla3 TSS. Female enriched RXRα peaks are indicated by arrows P1, P2, P3 and P4. Right-hand panel: Sexual dimorphic RNA levels of Pnpla3 were confirmed by qPCR (n = 5–6) (see also Fig. S2, S3A–G, S7 and S8). *p<0.05

RXRα and Pol2 binding to a male-enriched (Cyp7b1), and female-enriched gene (Pnpla3; Fig. 4) highlight the power and utility of the RXRα+Pol2 assay approach. Note the ability of the algorithm to identify genes with strong RXRα-associated gender-specific binding and transcription, even in the presence of additional RXRα binding on liver chromatin of the opposite gender (e.g. several adjacent male RXRα peaks in Pnpla3, Fig. 4 + Fig. S2). RNA quantification from mouse liver samples confirmed the strong sexually-dimorphic expression of these genes (Fig. 4C). Maps of other genes with RXRα and Pol2 scores >6 that underwent further analyses are shown in Fig. S3 (i.e. Chip-Seq maps and RNA quantification for Elov6, Scd1, Lpin1, Elovl3, Hsd3b5, Sult3a1). Sexually-dimorphic expression of the Pnpla3 gene, which is known to be regulated by Srebp1c [44], [45] (but not known to be regulated by RXRα heterodimers) with a polymorphism (I148M) strongly associated with fatty liver disease, has not been reported beforehand. The consequences of this polymorphism in relation to PNPLA3 function and hepatic lipid handling are not fully known [46].

### Incorporation of gene expression levels into the ChIP-Seq RXRα+Pol2 analyses

**Figure 5:**
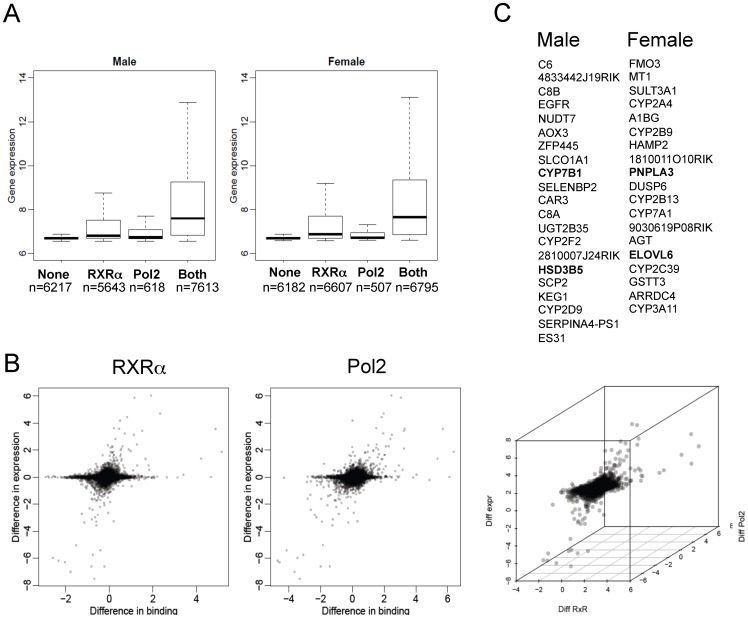
Correlation of gender differential RXRα, Pol2 and RNA expression levels. A) Correlation of RXRα and Pol2 binding with gene expression levels. B) Scatterplot representation of gender differential RXRα binding with gender differential gene expression on the left-hand side, and scatterplot representation of gender differential Pol2 binding with gender differential gene expression on the right-hand side. C) Genes with a gender specific positive correlation between RXRα binding Pol2 binding and changes in RNA levels (see also Fig. S4A–B and Table S6A–H in File S1).

RNA expression levels in male and female mouse livers were determined by microarray analyses and correlated with RXRα and Pol2 binding strengths. This analysis revealed that the RNA expression levels were, in aggregate, highest when both RXRα and Pol2 binding were present (Fig. 5A). In addition, binding of RXRα and Pol2 surrounding the TSS was most strongly correlated with higher gene expression levels in both male and female mouse liver (Fig. 5B). Differentially-expressed male-female genes using RNA levels (employing a cutoff of a 1.5 fold change in expression in either direction), resulted in 846 male-enriched and 613 female-enriched genes. Correlation of strong RXRα binding sites (score >6) to sexually-dimorphic RNA levels, identified 142 genes (395 RXRα peaks) in male livers, and 117 genes (305 RXRα peaks) in female livers (Fig. S4A;Table S5A–B in File S1), many of which were previously identified as gender-specific [11], [47], [48]. Further analysis correlated strong sexually-dimorphic Pol2-binding with RNA levels in 23 male-specific genes and 24 female-specific (Fig. S4B, Table S5C–D in File S1). Finally, incorporating all 3 analyses (RXRα peaks with score >6, Pol2 binding score >6, and RNA levels FC>1.5, p<0.1) with regards to gender-specific enrichment, 21 genes were identified for male and 19 for females (Fig. 5C, Table S5E–F in File S1). Of note, there is significant enrichment of lipid and steroid-processing genes when RNA levels are incorporated into the RXRα +Pol2 score, including PNPLA3 in female livers and Cyp7b1 in male livers, overall validating the utility of the RXRα+Pol2 score as a means of identifying genes with strong RXRα binding sites that are likely to be highly regulated by RXRα-containing heterodimers. We next sought to validate such ChIP-Seq based analyses and their potential to identify RXRα–regulated genes with in vivo studies using the potent RXRα agonist, LG268.

### Responses of mouse livers to the RXRα agonist LG268

**Figure 6:**
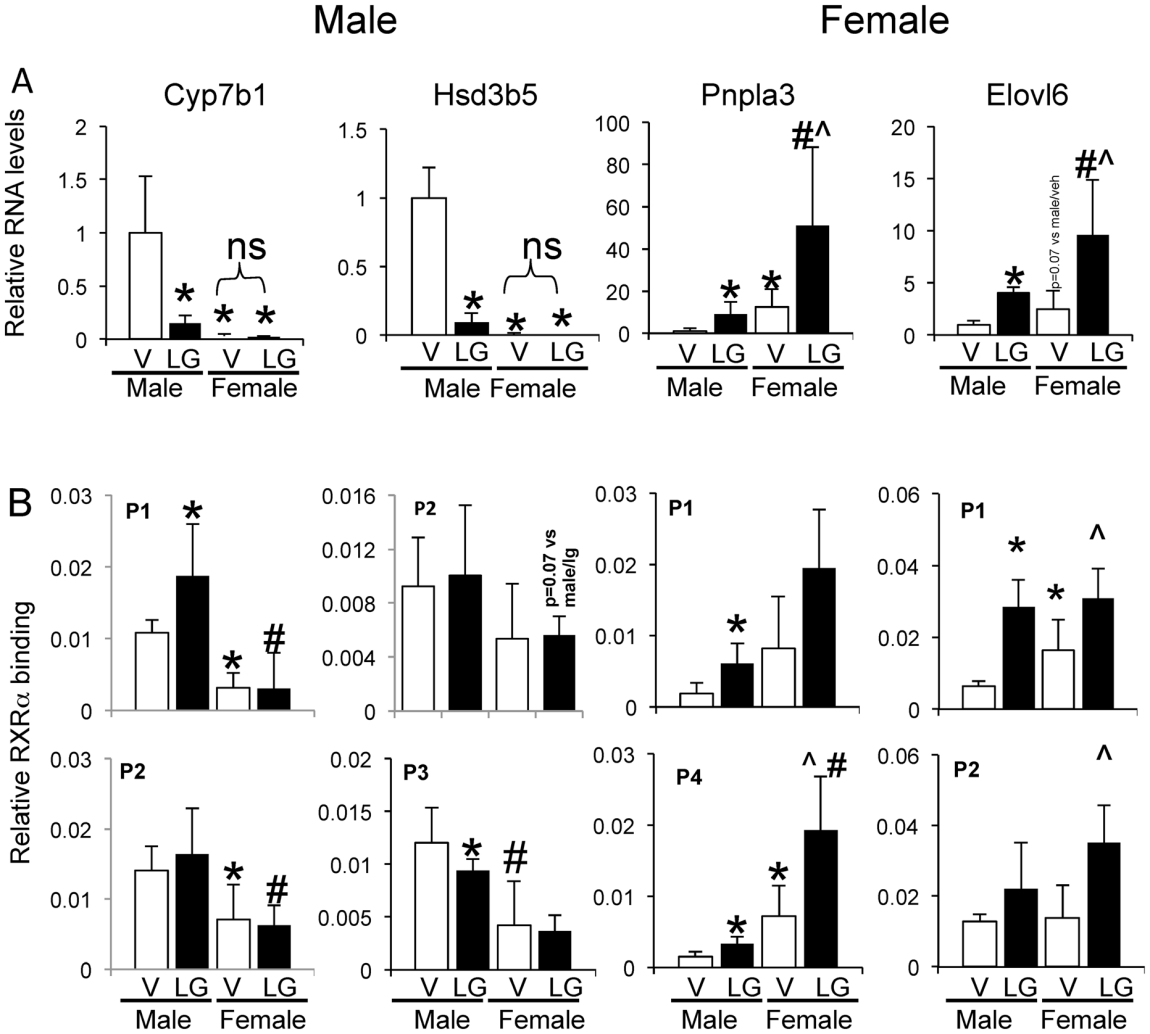
Gender differential responsiveness to RXRα activation in vivo. Male and female mice were gavaged once daily for 5 days with the RXRα ligand LG268. A) Relative RNA levels in male and female mouse liver as determined by QPCR in response to RXRα activation by LG268 for selected male and female enriched genes. B) RXRα occupancy in response to RXRα activation on selected male and female enriched genes (see also Fig. S5 and S6). *p<0.05 vs male/veh; ?p<0.05 vs female/veh; #p<0.05 vs female/LG268.

In order to determine gender and gene-specific capability to respond to an RXRα ligand, male and female mice were given the RXRα ligand (rexinoid) LG268 daily for 5 days by gavage feeding, followed by analysis of RXRα binding by ChIP-qPCR and RNA quantification. RXRα binding to upstream regions of Pnpla3 and Elovl6 genes was increased (1.8–4.7-fold) in male mice when given the ligand, with an even greater increase in binding in LG268-treated female mice (3.0–10.2-fold; Fig. 6B). However, binding of RXRα in response to LG268 was not changed for the male gene binding sites for Cyp7b1 and Hsd3b5 (Fig. 6B). LG268 treatment increased RNA levels of Pnpla3 in both male and female livers ∼9–12 fold, and given the substantially greater expression in females, led to an overall 50-fold higher expression of Pnpla3 RNA in LG268-treated female livers than males treated with vehicle alone (Fig. 6A). A similar pattern of changes was noticed for Elovl6, Interestingly, RNA levels of male-specific genes Cyp7b1 and Hsd3b5 were repressed by LG268 (Fig. 6A). Liver triglyceride (TG) levels increased 2-fold in male mice given LG268, bringing the male liver TG content in line with the basally higher TG content of female livers (Fig. S5), indicating physiological relevance of our findings of hepatic TG levels in response to RXRα activation.

Moreover, LG268 increased RXRα binding site occupancy in the sentinel female-specific genes (Pnpla3, Elovl6) in both male and female mouse livers, more so in females than males (Fig. 6),while there were variable and more modest effects of LG268 on RXRα binding in male-enriched sites. Whether or not this is a consequence of a greater abundance of RXRα in female than male liver nuclei remains to be determined (Fig. 1D).

## Discussion

In this study we identified genome-wide binding sites for RXRα and Pol2 in mouse liver and discovered sexually-distinct binding patterns in males and females that were enriched in sterol and lipid processing genes. To our knowledge, the number of binding sites determined in this study for RXRα in both genders (>45,000) is the most for any known transcription factor, correlating with ∼50% of all mouse genes, and 32% of liver-expressed genes (based on RNA levels and probe sets of total mouse genome present in our microarray dataset). Given the extensive binding in mouse liver chromatin, RXRα as an essential heterodimer partner to Type 2 NRs, should be considered as a natural focus for exploring the core mechanisms underlying the metabolic machinery, and response to the intracellular environment of the metabolically-active hepatocyte. The vast number of binding sites found for RXR α in this study correlates well with its broad-based function in liver due to heterodimeric partners and the associated large number of binding sites with each of the known dozen partner NRs expressed in liver [49]. Overlapping sites have been found for FXR, PPARα, LXRα [28], [29] indicating many of these sites are functional. Functionality and partner-identification of the remaining sites will have to be determined in future studies that incorporate ChIP-SEQ with ligand and mechanistic analyses.

RXRα binding to chromatin was enriched in male mouse liver for 1945 genes and 1214 genes in females which included both new and previously-identified hepatic gender specific and enriched binding sites and genes. Though the main physiological processes that were found associated with RXRα binding were similar between genders, including lipid, sterol and drug related metabolism processes, different genes within these same process/pathways were enriched for RXRαbinding in each gender, indicating potential differential roles for RXRα regulation of these pathways. Moreover, a new analytical tool was developed that incorporated RXRαbinding strength with Pol2-binding strength of an assigned closest gene. Using this analysis, a surprisingly small and metabolically-relevant list of genes was identified–44 male and 43 female enriched genes, the majority involved in drug or lipid metabolism. The gender-discrepancy in lipid processing genes and RXRα binding was unexpected and identified RXRα dependent sexually-dimorphic binding as a major player in lipid metabolism.

Effective handling of hepatic lipids likely directs whether or not an organism develops pathophysiological consequences of fat accumulation, with direct links to the most common form of liver disease in humans – Non-alcoholic fatty liver disease (NAFLD) [50]. With the worldwide increase in obesity, NAFLD is a leading cause of chronic liver disease, affecting about 20–25% of the general population worldwide, 20–30% of which may progress to cirrhosis [51]–[55]. Lipid metabolism is gender-dimorphic [56], and NAFLD appears to be more prevalent in men than women, both in adults and in pediatric populations [57]–[61]. In mice, gender differences in hepatic lipid composition was shown, and livers of female mice contained twice the amount of total lipids compared to male mouse livers [62]. Sex-differential expression in metabolism-related genes in response to high fat diet [63], [64] have been observed as well and it appears that female livers have a superior capability of handling of changes in lipid concentrations and maintaining homeostasis. We found higher binding of RXRα to female hepatic chromatin for the essential lipid processing genes including Pnpla3, Lpin1, RGS16_ENREF_119, Elovl6, SCD1 and Fasn (see below), supporting a central role for RXRα in mediating these differences. In addition, LG268-activated RXRα showed higher induction of lipid-related gene expression in female compared to male mice, but without higher induction of hepatic TG levels, consistent with the improved capability of handling lipids in female mice. PNPLA3 (adiponutrin) is a patatin-like phospholipase [65], [66]. Recently its function has been elucidated to be LPAAT activity [67], mediating one of the steps in the TG synthesis pathway but loss of *Pnpla3* in mice did not cause fatty liver [45], [68], [69]. Pnpla3 RNA expression is regulated by nutritional factors, including feeding and fasting and intra-hepatic lipid status [44], [70], [71]. Consistent with higher female hepatic lipid levels, we show here that Pnpla3 has female-dominant RXRα binding and RNA expression in liver, with enhanced responsiveness to RXRα activation, suggesting a major role for RXRα in mediating this gender lipid difference. Pnpla3 gene expression is directly regulated by Srebp1c [44], [45], and indirectly by the LXR/RXRα heterodimer acting on Srebp1c. Our ChIP-Seq and ChIP-QPCR data reveal also direct regulation by RXRα heterodimers. No gender or LG268 induced differences in RNA levels for Srepb1 were detected, despite a tendency for higher RXRα and Pol2 binding to female liver chromatin (Fig. S6). Numerous recent studies have established that the rs738409 C>G polymorphism encoding for the PNPLA3 I148M variation affects hepatic TG content and is more prevalent among NAFLD patients than healthy controls [39], [40], [72]. In addition, this polymorphism is associated with severity of both steatosis and fibrosis in NASH. Lpin1, which was also identified in our analysis to be female enriched in RXRα and Pol2 binding (Fig. 3), regulates a step in TG synthesis after Pnpla3 and serves as coactivator for certain PPAR/RXRα regulated genes [73]. Our data show strong direct gender-differential binding of RXRα to Lpin1 upstream regions. Upstream of TG synthesis, Elovl6 is involved in elongation of saturated fatty acids (FA) from C12–16 to C18 [74], [75], and its role in FA-synthesis is in conjugation with Fasn and Scd1. Elovl6 transcription is indirectly activated by the RXRα/LXRα heterodimer via activation of Srebp1c [75], [76], but our data indicate direct regulation by RXRα and partners via multiple binding sites as well, with an additional newly identified gender-specific regulatory role for RXRα. Scd1 is transcriptionally regulated by multiple partners of RXRα, including LXRα and PPARα [77], and multiple RXRα binding sites were identified in our study, but regulation was not previously known to be RXRα-mediated and gender differentially affected. RGS16 is involved in regulating energy homeostasis by coupling hepatic glucose production with rate of FAO [78], and Rgs16-Tg mice developed fatty liver during prolonged fast [79], and gender differential expression as well as regulation by RXRα has not been shown before. Overall, the roles found here for RXRα-containing heterodimers are readily extended to known therapeutic effects of ligands under study for these same pathways that target RXRα's partners- PPARs, LXRs, and FXR. The consequences to RXRα-partner binding and function after heterodimer partner ligand treatment, or treatment with a rexinoid in combination with a partner ligand, is important and in need of future study.

Previous studies in human and rodent liver have identified sex-dependent differences in hepatic gene expression involved in diverse hepatic processes, including inflammatory responses, lipid metabolism, and steroid and drug metabolism [11], [12], [80], [81], which were partially replicated in our study. In addition, by combining ChIP-Seq with RNA microarray analyses, this was expanded with findings of novel sex differences implicating a role for RXRα. Previous studies have also shown that gender differences in Growth Hormone (GH) pituitary secretion patterns play a major role in determining sexual dimorphic hepatic gene expression [15], [17], [47], [82], [83], however it does not explain all aspects. An essential factor mediating the hepatic gender-specific effects of GH is the downstream transcription factor Stat5b, and its absence in male liver induced feminized hepatic gene expression [16]. Although Stat5b is a significant factor, other factors have been identified as well to play a role in sexual dimorphic hepatic gene expression, including transcription factors enriched in female liver Cux2, Trim24, Tox [18] and Bcl6 in male mouse liver [84]. In addition, some partners of RXRα were shown to have gender differential effects under certain conditions [48], [85] [25]. Cai et al [86] showed a role for RXRα signaling in hepatic gender differences for expression levels of several CYP genes; changes which were dependent on or influenced by sex hormones. Gender-dimorphism was also shown for hepatic PPARα [20], [85], CAR [87], [88], and HNF4α [89], [90]. Additional factors in hepatic gender differential expression are the sex hormones and their receptors, which are also a part of the NR family (Class I). Crosstalk has been shown between GH, NRs, sex hormones and their downstream signaling pathways [25], affecting hepatic sexual dimorphic expression of genes with roles in hepatic lipid metabolism. How all these differences in NR expression and function relate to our findings of differential chromatin occupancy of RXRα in male and female mouse livers will require similar sets of studies performed for the various NRs, especially ligand treatment of specific RXRα heterodimerization partners. Recently Zhang et al [26] identified all sexual dimorphic binding sites for Stat5b in male and female mouse liver. Comparing these data to our results for RXRα showed that ∼28% of all genes (>12 k) in male mouse liver with RXRα binding sites also contained Stat5b binding sites, and >92% of the genes with Stat5b binding sites were identified to also have RXRα binding sites (Fig. S7, Table S6A–B in File S1). Additional comparisons show a high correlation for a major part of gender-specific RXRα and Stat5b binding sites (Fig. S7, Table S6A–B in File S1 ). As pointed out by others [26], [29], [91], this indicates that there is likely a considerable overlap and coregulation between TFs on the same genes, even with overlapping binding sites. For example, in our study Stat5b and RXRα binding at the same chromosomal location was found for Cyp7b1, Hsd3b5 (Fig. S7), but not for the female genes Pnpla3 and Sult3a1, indicating complex regulation of sex differential gene expression and roles for cooperation and interaction of multiple transcription factors. Whether or not there is true cooperativity between Stat5b and RXRα heterodimers in NR ligand responsive gene expression in liver remains to be determined.

All together, these gender-specific binding patterns and differental ligand responsiveness may underlie some of the gender-specific “idiosyncratic” effects of drugs and the propensity to adverse events noted in humans. In conclusion these data indicate that RXRα is not only one of the most widely-distributed transcriptional regulators in mouse liver, but is engaged in determining sexually-dimorphic expression of several key sterol and lipid processing genes. These studies have implications for a deeper understanding of the roles for RXRα and heterodimer partner regulation of hepatic gene expression, with implications for the efficacy and adverse event profiles of NR ligands with potential gender-specific responses.

## Supporting Information

Figure.S1 **Chromosomal distribution of RXRα and Pol2 binding sites in male and female liver.** A) Chromosomal distribution of RXRα (left-hand panel) and Pol2 binding (right-hand panel) sites in male (top) and female liver (bottom). B) Number of RXRα (left-hand panel) and Pol2 (right-hand panel) binding sites per chromosome in male (top) and female liver (bottom) (see also Figure 1), showing significantly higher number of sites observed on chromosome 11 compared to expected and significantly lower on the X-chromosome.
(TIF)

Figure.S2 **RXRα and Pol2 binding sites for Akr1d1 in male and female mouse liver.** Screenshot of *Akr1d1* as an example of a gene with peaks identified with both male and female enriched RXRα peaks (see also Figure 4).
(TIF)

Figure.S3 **RXRα and Pol2 binding sites for sexually dimorphic genes in male and female mouse liver.** A) *Elovl6*, B) *Scd1*, C) *Lpin1*, D) *Elovl3*, E) *Hsd3b5*, F) S*ult3a1* and G) *Rsg16*. Top panels of each sub-figure shows screenshots of sexually dimorphic RXRα peaks (indicated by arrows) and Pol2 binding. Lower panel of each sub-figure shows RNA levels for each gender as determined by realtime qPCR for with and without response to the synthetic RXRα ligand LG268 (see also Figure 4 and Figure 6).
(TIF)

Figure S4 **Sexually dimorphic RXRα and Pol2 binding sites correlated to gender differential changes in RNA levels.** A) Scatterplot correlation of gender differential RXRα binding sites with score >6 and gender differential changes in RNA levels for male and female mouse liver. B) Scatterplot correlation of gender differential Pol2 binding sites and gender differential changes in RNA levels for male and female mouse liver (see also Figure 4).
(TIF)

Figure S5 **Hepatic triglyceride levels in male and female mouse liver.** Triglyceride levels in male and female livers in response to ligand activated RXRα (see also Figure 6). Mice were gavaged for 5 days once daily with the synthetic RXRα ligand LG268 (30 mg/kg). *p<0.05 vs male/veh.
(TIF)

Figure S6 **RXRα binding, Pol2 binding and RNA levels of Srebp1c in male and female mouse liver.** Top panel shows screenshot of RXRα binding and Pol2 binding to *Srebp1* in male and female mouse liver. Lower panel shows RNA levels of Srebp1c in response to RXRα activation by the synthetic RXRα ligand LG268 in male and female mouse liver (see also Figure 6).
(TIF)

Figure S7 **Overlap of genes with binding sites for RXRα and Stat5b in a gender-specific manner.** A–D) Venn diagrams showing overlap of number of sexual-dimorphic genes with binding sites identified for RXRα and for Stat5b. Stat5b data were obtained from (Zhang et al., 2012) (see also Figure 4).E–H) Screenshots of overlap of gender-specific binding sites for RXRα and Stat5b at the same chromosomal location for Cyp7b1 (E) and Hsd3b5(F), but not for Pnpla3 (G) and Sult3a1 (H) (see also Figure 4)
(TIF)

File S1 This file contains Tables S1A through S6. Table S1A, Number of RXR and Pol2 reads and peak in male and female mouse liver: related to Figure 1 Table S1B, Male RXRa peak height >300; related to figure 1. Table S1C, Female RXR peak height >300; related to figure 1. Table 1D, RXRa peak distribution. Table S2A, ontology biological pathways; related to Figure 2. Table S2B, Kegg pathways; related to Figure 2. Table S3A, Male preferential RXR-binding sites: Male RXR peaks, RXR score >6; related to Table 1. Table S3B, Female preferential RXR-binding sites: Female RXR peaks, RXR score >6; related to Table 1. Table S3C, Male preferential RXR-binding sites: Male RXR only peaks, RXR score >6; related to Table 1. Table S3D, Female preferential RXR-binding sites: Female RXR peaks only, RXR score >6; related to Table 1. Table S3E, Female-Male overlap, RXR score >6; related to Table 1. Table S3F, Male preferential transcribed genes: Pol2 score >6;related to Table 1. Table S3G, Female preferential transcribed genes: Pol2 score >6;related to Table 1. Table S4A, Male preferential RXR-binding sites: Male RXR score >6 & Pol2 score>6; related to figure 5. Table S4B,Female preferential RXR-binding sites: Female RXR score >6 & Pol2 score>6; related to figure 5. Table S4C, ontology biological pathways; related to figure 5. Table S4D, Kegg pathways; related to figure 5. Table S5A, RXR vs RNA RXR>6-RNA F FC>1.5 ttest<0.1; related to Figure 6. Table S5B, RXR vs RNA RXR>6-RNA F FC>1.5 ttest<0.1; related to Figure 6. Table S5C, Pol2 vs RNA Pol2>6-RNA M FC>1.5 ttest<0.1; related to Figure 6. Table S5D, Pol2 vs RNA Pol2>6-RNA F FC>1.5 ttest<0.1; related to Figure 6. Table S5E, RXR-Pol2-RNA M; related to Figure 6. Table S5F, RXR-Pol2-RNA F; related to Figure 6. Table S6A, Comparison RXRa and Stat5b genes; Male- related to figure 4. Table S6B, Comparison RXRa and Stat5b genes; Female – related to figure 4.
(XLSX)
