# Supplementary figures and images for: Sexually Dimorphic Genome-Wide Binding of Retinoid X Receptor alpha (RXRα) Determines Male-Female Differences in the Expression of Hepatic Lipid Processing Genes in Mice
Source: PLoS One. 2013 Aug 19;8(8):e71538. doi: 10.1371/journal.pone.0071538 (PMC3747242; doi:10.1371/journal.pone.0071538)

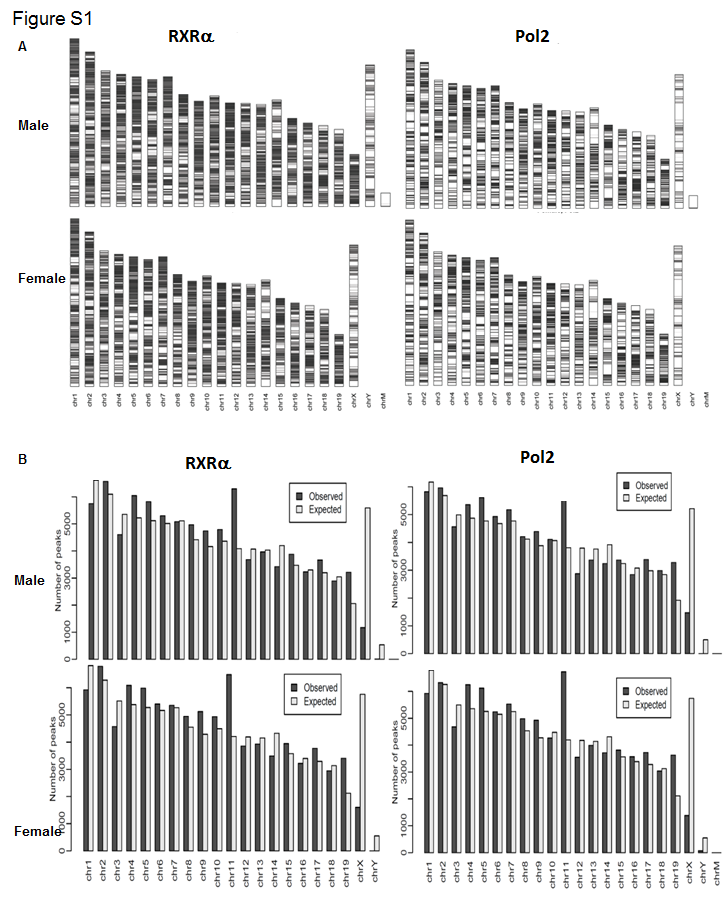

Supplement: Figure.S1 — Chromosomal distribution of RXRα and Pol2 binding sites in male and female liver. A) Chromosomal distribution of RXRα (left-hand panel) and Pol2 binding (right-hand panel) sites in male (top) and female liver (bottom). B) Number of RXRα (left-hand panel) and Pol2 (right-hand panel) binding sites per chromosome in male (top) and female liver (bottom) (see also Figure 1), showing significantly higher number of sites observed on chromosome 11 compared to expected and significantly lower on the X-chromosome. (TIF) [file pone.0071538.s001.tif]

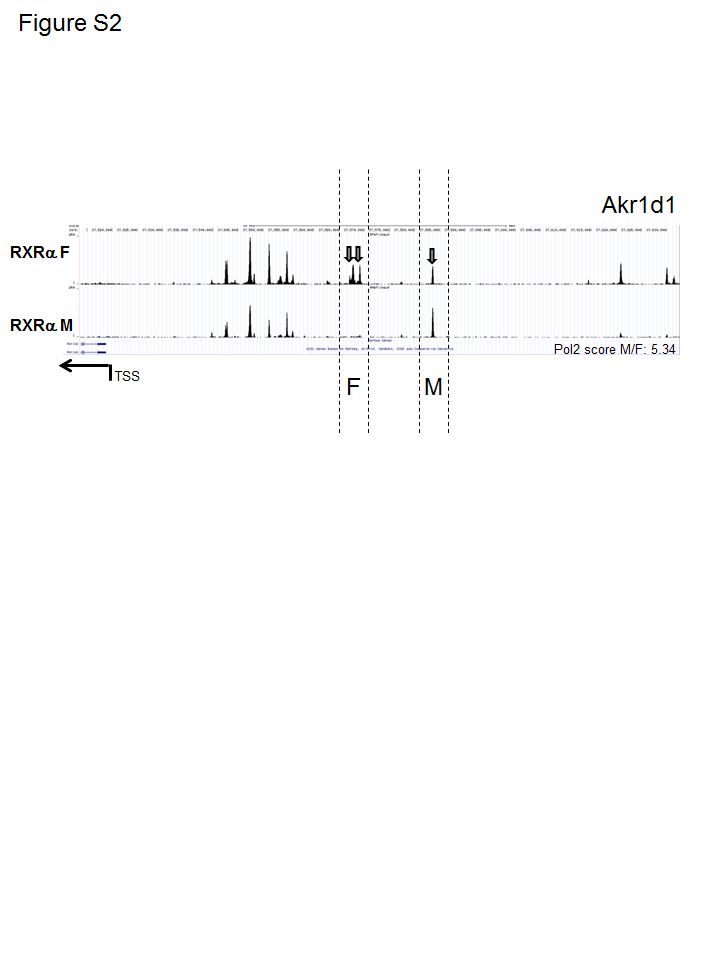

Supplement: Figure.S2 — RXRα and Pol2 binding sites for Akr1d1 in male and female mouse liver. Screenshot of Akr1d1 as an example of a gene with peaks identified with both male and female enriched RXRα peaks (see also Figure 4). (TIF) [file pone.0071538.s002.tif]

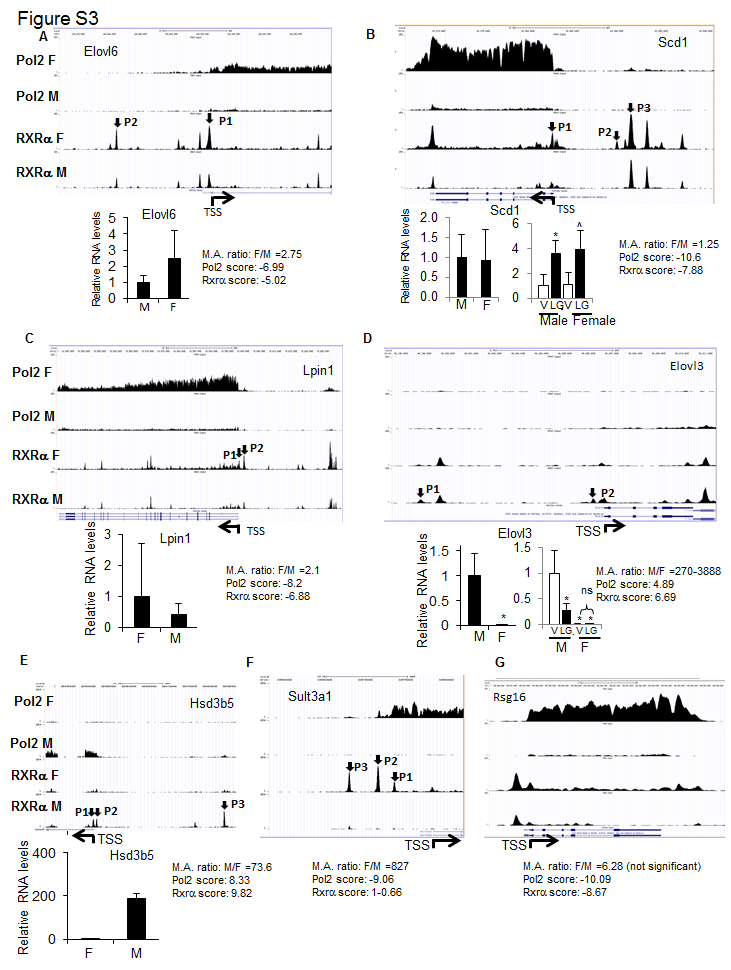

Supplement: Figure.S3 — RXRα and Pol2 binding sites for sexually dimorphic genes in male and female mouse liver. A) Elovl6, B) Scd1, C) Lpin1, D) Elovl3, E) Hsd3b5, F) Sult3a1 and G) Rsg16. Top panels of each sub-figure shows screenshots of sexually dimorphic RXRα peaks (indicated by arrows) and Pol2 binding. Lower panel of each sub-figure shows RNA levels for each gender as determined by realtime qPCR for with and without response to the synthetic RXRα ligand LG268 (see also Figure 4 and Figure 6). (TIF) [file pone.0071538.s003.tif]

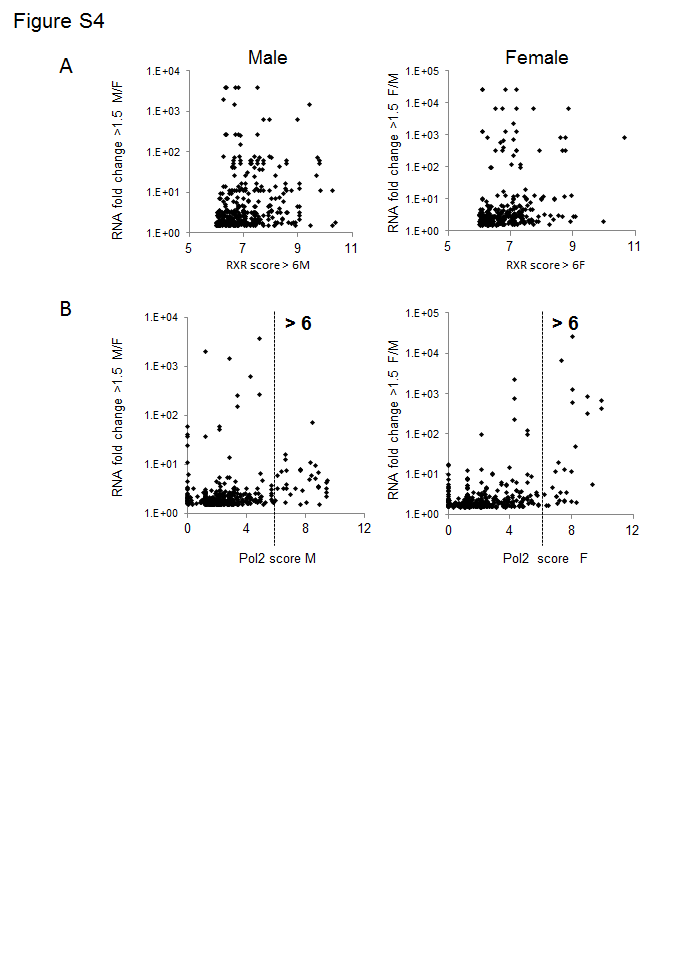

Supplement: Figure S4 — Sexually dimorphic RXRα and Pol2 binding sites correlated to gender differential changes in RNA levels. A) Scatterplot correlation of gender differential RXRα binding sites with score >6 and gender differential changes in RNA levels for male and female mouse liver. B) Scatterplot correlation of gender differential Pol2 binding sites and gender differential changes in RNA levels for male and female mouse liver (see also Figure 4). (TIF) [file pone.0071538.s004.tif]

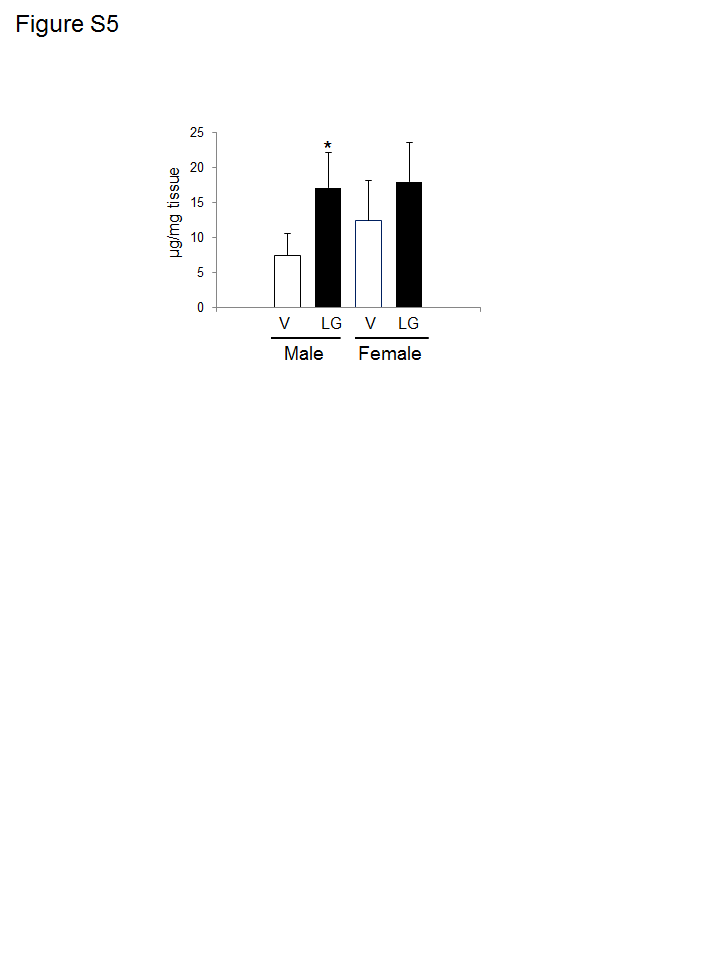

Supplement: Figure S5 — Hepatic triglyceride levels in male and female mouse liver. Triglyceride levels in male and female livers in response to ligand activated RXRα (see also Figure 6). Mice were gavaged for 5 days once daily with the synthetic RXRα ligand LG268 (30 mg/kg). *p<0.05 vs male/veh. (TIF) [file pone.0071538.s005.tif]

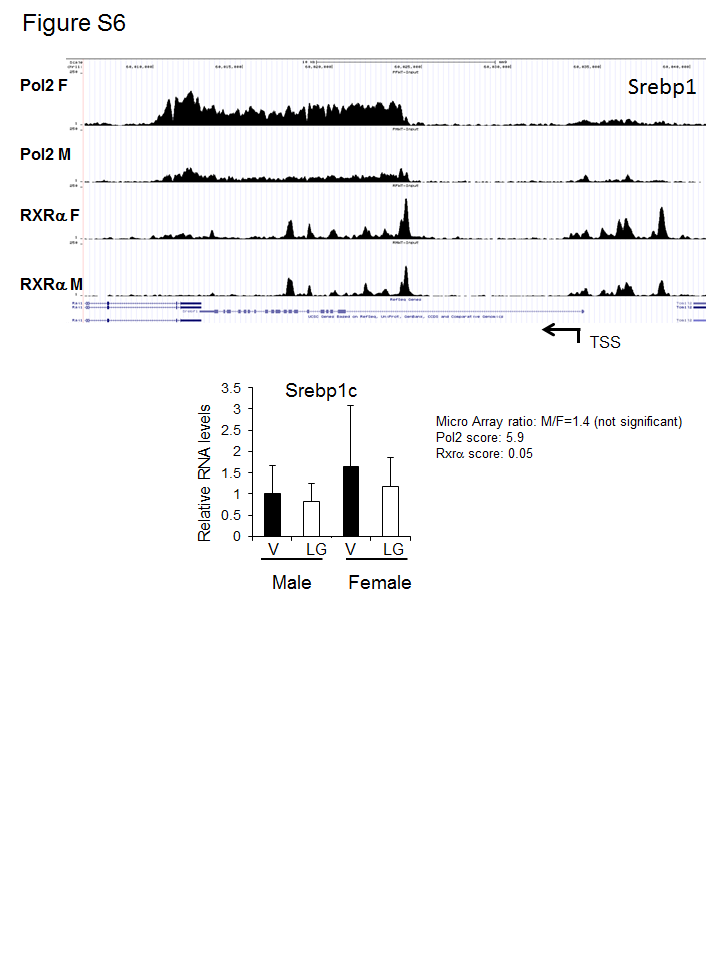

Supplement: Figure S6 — RXRα binding, Pol2 binding and RNA levels of Srebp1c in male and female mouse liver. Top panel shows screenshot of RXRα binding and Pol2 binding to Srebp1 in male and female mouse liver. Lower panel shows RNA levels of Srebp1c in response to RXRα activation by the synthetic RXRα ligand LG268 in male and female mouse liver (see also Figure 6). (TIF) [file pone.0071538.s006.tif]

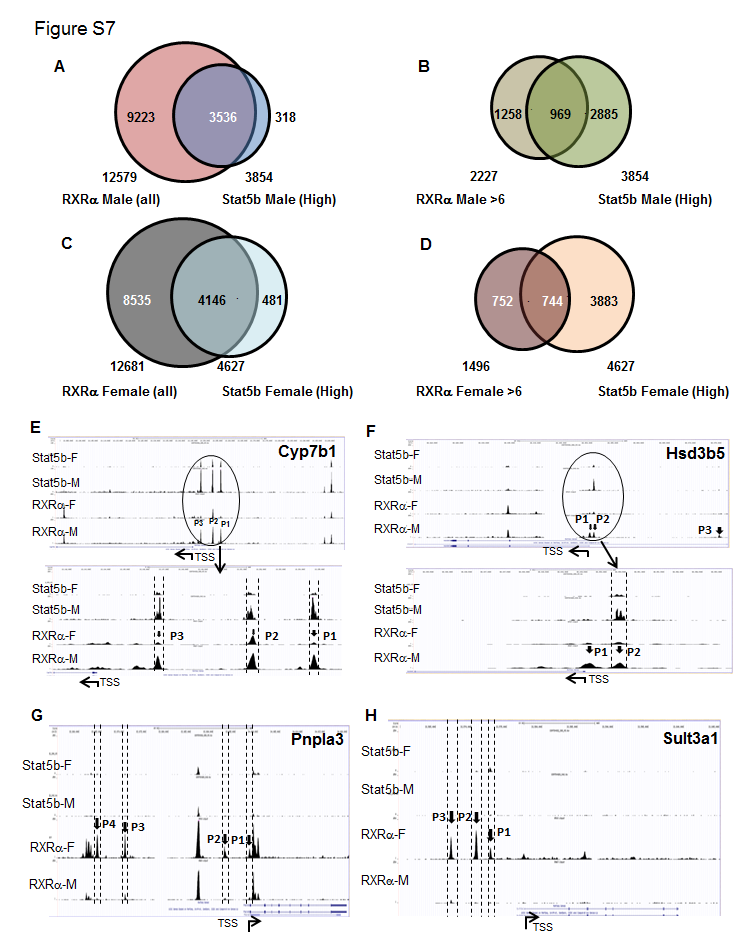

Supplement: Figure S7 — Overlap of genes with binding sites for RXRα and Stat5b in a gender-specific manner. A–D) Venn diagrams showing overlap of number of sexual-dimorphic genes with binding sites identified for RXRα and for Stat5b. Stat5b data were obtained from (Zhang et al., 2012) (see also Figure 4).E–H) Screenshots of overlap of gender-specific binding sites for RXRα and Stat5b at the same chromosomal location for Cyp7b1 (E) and Hsd3b5(F), but not for Pnpla3 (G) and Sult3a1 (H) (see also Figure 4) (TIF) [file pone.0071538.s007.tif]
